# Racial and Ethnic Disparities in Gynecologic Carcinosarcoma: A Single-Institution Experience

**DOI:** 10.3390/cancers15194690

**Published:** 2023-09-23

**Authors:** Kristina E. Mercado, Nora M. Badiner, Canty Wang, Laura Denham, Juli J. Unternaehrer, Linda J. Hong, Yevgeniya J. Ioffe

**Affiliations:** 1 Department of Gynecology and Obstetrics, Loma Linda University Medical Center, Loma Linda, CA 92354, USA; 2 Division of Gynecologic Oncology, Department of Gynecology and Obstetrics, Loma Linda University Medical Center, Loma Linda, CA 92354, USA; 3 Department of Pathology, Loma Linda University Medical Center, Loma Linda, CA 92354, USA; 4 Division of Biochemistry, Department of Basic Sciences, Loma Linda University, Loma Linda, CA 92354, USA

**Keywords:** gynecologic carcinosarcoma, malignant mixed Mullerian tumor, incidence, overall survival, ethnic disparity, racial disparity

## Abstract

**Simple Summary:**

Carcinosarcoma is a rare and aggressive gynecologic cancer that may arise in any of the organs of the female reproductive tract. Most commonly, gynecologic carcinosarcoma is of uterine origin and was previously known as malignant mixed Mullerian tumor. Uterine carcinosarcoma accounts for less than 5% of all uterine malignancies. Because of the rarity of this diagnosis, gynecologic carcinosarcomas have been understudied. We aimed to determine the incidence, treatment course, and quantitate treatment outcomes via progression-free survival (PFS) and overall survival (OS) of gynecologic carcinosarcoma in a primarily minority patient population.

**Abstract:**

We aimed to determine the incidence, treatment regimen, and treatment outcomes (including progression-free survival and overall survival) of gynecologic carcinosarcoma, a rare, aggressive, and understudied gynecologic malignancy. This retrospective review included all patients with gynecologic cancers diagnosed and treated at a single tertiary care comprehensive cancer center between January 2012 and May 2021. A total of 2116 patients were eligible for review, of which 84 cases were identified as carcinosarcoma: 66 were uterine (5.2% of uterine cancers), 17 were ovarian (3.6% of ovarian cancers), 1 was cervical (0.28% of cervical cancers), and 1 was untyped. Of the patients, 76.2% presented advanced-stage disease (stage III/IV) at the time of diagnosis. Minority patients were more likely to present with stage III/IV (*p* < 0.0001). The majority of patients underwent surgical resection followed by systemic chemotherapy with carboplatin and paclitaxel. The median PFS was 7.5 months. Of the patients, 55% were alive 1 year after diagnosis, and 45% were alive at 5 years. In the studied population, minorities were more likely to present with more advanced disease. The rate of gynecologic carcinosarcomas was consistent with historical reports.

## 1. Introduction

Carcinosarcoma, previously known as malignant mixed Mullerian tumor (MMMT), is a rare, aggressive, metaplastic carcinoma that can arise in the uterus (UCS), ovary (OCS), or cervix (CCS). Rare carcinosarcomas arising in the fallopian tubes and peritoneum have also been reported [1]. Given the rarity of the diagnosis and its aggressive nature, it has been difficult to estimate the true incidence of the disease. UCS accounts for less than 5% of all uterine malignancies, and incidence in the United States has been estimated to range from 1 to 4 per 100,000 women [2]. Analysis of the Surveillance Epidemiology and End Results (SEER) database suggests that the incidence of UCS has increased over time, from 2.2 per 1,000,000 in 2000 to 5.5 per 1,000,000 in 2016 [3]. Compared with UCS, OCS is even more rare, accounting for between 1 and 4% of ovarian cancer diagnoses in the United States [4]. SEER database analysis results in an incidence of 0.6 per 1,000,000 for OCS and 0.1 per 1,000,000 for CCS [3].

Carcinosarcomas are biphasic tumors with both an epithelial and a mesenchymal component [5]. There are several theories regarding the pathogenesis of this aggressive disease. The currently accepted hypothesis is known as “combination theory” [6] or the “monoclonal hypothesis” [7]. This theory proposes that both the epithelial and mesenchymal components arise from a single multipotent stem cell. The epithelial component may be an admixture of serous, endometrioid, or squamous carcinoma. The mesenchymal component may be homologous cartilaginous or skeletal muscle components and, more rarely, osteosarcoma or liposarcoma. Or, it may be heterologous, containing multiple tissue types [8]. The relative proportions of epithelial and mesenchymal components vary widely from tumor to tumor. Therefore, it is recommended that pathology reports include the percentages and types of the epithelial and mesenchymal elements present [9].

According to the National Comprehensive Cancer Network (NCCN) guidelines, primary treatment of UCS and OCS typically consists of extirpative surgery with total hysterectomy, bilateral salpingo-oophorectomy, peritoneal lavage for cytology, and omental and peritoneal biopsies, with a goal of achieving complete cytoreduction to no gross residual disease [10,11]. Following primary surgical staging and debulking, adjuvant carboplatin and paclitaxel chemotherapy is recommended for both UCS and OCS, regardless of stage [10,11]. Radiation is recommended for UCS in cases where the sarcomatous component comprises >50% of the tumor [10]. Several studies have shown improved progression-free survival with combination therapy [12], but due to the rarity of carcinosarcoma, it is difficult to perform large-scale clinical trials. Ifosfamide-paclitaxel [13] and carboplatin-paclitaxel [14] have both been shown to improve survival. Tumor-directed radiation therapy may include external beam radiation therapy and/or vaginal brachytherapy [15]. Pelvic radiation therapy has been shown to decrease the rate of local recurrence in early-stage disease [15].

Given the rarity of gynecologic carcinosarcoma, we aimed to describe its incidence, racial distribution, stage at presentation, and treatment course, and quantify progression-free survival (PFS) and overall survival (OS).

## 2. Materials and Methods

After Institutional Review Board approval was obtained, a single-institution retrospective chart review was conducted. The charts of patients newly diagnosed with gynecologic cancer from January 2012 to May 2021 were reviewed for final pathologic diagnosis to identify the cases of carcinosarcoma. Exclusion criteria included untyped carcinosarcoma, diagnosis of another malignancy with complete remission less than two years at the time of carcinosarcoma diagnosis, ongoing chemotherapy treatment for a diagnosis of other malignancy, and synchronous diagnosis of other malignancy. In this study, 1 patient was excluded due to the presence of an untyped carcinosarcoma, leaving 83 patients for inclusion in the study.

The electronic medical records were reviewed for diagnosis, demographics, pathologic tumor characteristics, stage at the time of diagnosis, clinical treatment course, and treatment outcomes. Demographic data abstracted included age, self-identified race/ethnicity, and body mass index. Pathologic data abstracted included tumor type. Treatment data were also abstracted, including stage at diagnosis, initial intervention performed (surgical, chemotherapy, or radiation), subsequent treatment modality, and outcomes. 

Outcome measures studied included median progression-free survival (PFS) and median overall survival (OS) for each cancer type (UCS, OCS, CCS). Progression-free survival (PFS) was defined as the time interval from the date of diagnosis to the date of detection of local, regional, or distant recurrence. Overall survival (OS) was defined as the time interval between the date of diagnosis and the date of last follow up or death from any cause. Incidence was calculated for carcinosarcoma overall, as well as by disease organ. Survival rates were calculated from the data. 

The median overall survival of patients by stage at diagnosis, race/ethnicity, lymph node involvement, and lympho-vascular space invasion were compared via a Mann–Whitney U test and a Kruskal–Wallis test, where appropriate. Patients undergoing surgery followed by adjuvant treatment were analyzed to examine the effects of adjuvant therapy on survival outcomes, comparing those who underwent chemotherapy alone with those undergoing combined chemoradiation. After categorization by adjuvant treatment type, a chi-square test was used to compare the baseline characteristics of the two groups. The Kaplan–Meier method was then used to construct survival curves comparing PFS and OS between the two adjuvant treatment groups. The median overall survival among patients of different stages at diagnosis was compared by race/ethnicity with the chi-squared test. 

## 3. Results

### 3.1. Incidence of Carcinosarcoma

A total of 2116 patients treated for invasive gynecologic malignancy during the study period of interest were reviewed: 1278 patients had a diagnosis of uterine malignancy, 476 of ovarian malignancy, and 362 of cervical malignancy. A total of 84 patients with carcinosarcoma were identified (Table 1). One patient was excluded from further analysis due to untyped carcinosarcoma, leaving eighty-three evaluable patients in the final study population. A total of 66 cases of uterine carcinosarcoma (UCS) were identified, accounting for 5.2% of all uterine malignancies. Of the patients with ovarian cancer eligible for review, 17 had carcinosarcoma of the ovary (OCS), representing 3.6% of ovarian malignancy cases. There was a single patient diagnosed with cervical carcinosarcoma (CCS), constituting 0.28% of cervical cancers. There was no consistent trend seen in the incidence of carcinosarcoma cases over time (Table 2), though there were noticeable drops in the number and incidence of UCS cases in 2019 and 2020.

### 3.2. Demographics

Demographic data are presented in Table 3. The median age at diagnosis for all carcinosarcomas was 68 years. Patients with uterine carcinosarcoma were older (median age of 69 years) than patients with carcinosarcoma of other gynecologic organs (those with ovarian cancer had a median age of 59 years; the single cervical patient was 58 years old; *p* = 0.00885). A total of 44.6% of patients self-identified as racial/ethnic minorities: 16.9% as Black/African American, as 22.9% Hispanic/Latina, and 4.8% as Asian/Pacific Islander. As expected, given the median age of all patients with carcinosarcoma in this study, 90.4% of patients were postmenopausal. The majority of patients were overweight or obese (67.4%).

**Figure 1:**
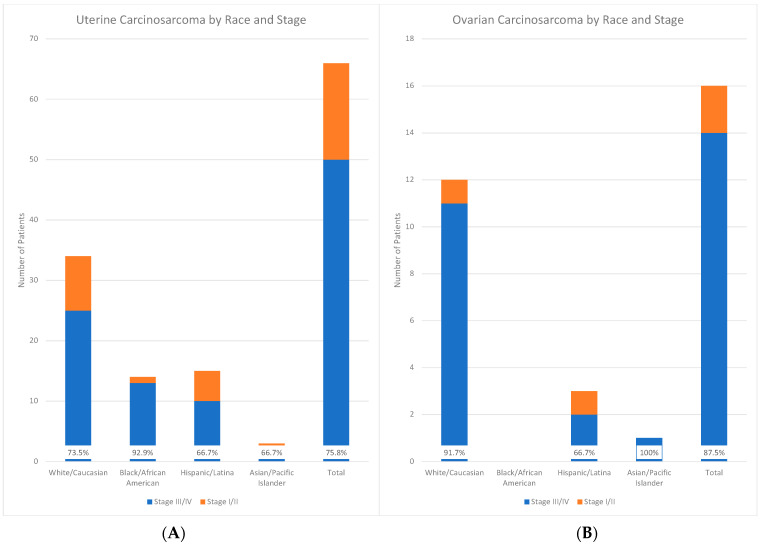
Graphical depiction of uterine (**left**, **A**) and ovarian (**right**, **B**) carcinosarcoma by race/ethnicity and stage.

**Figure 2:**
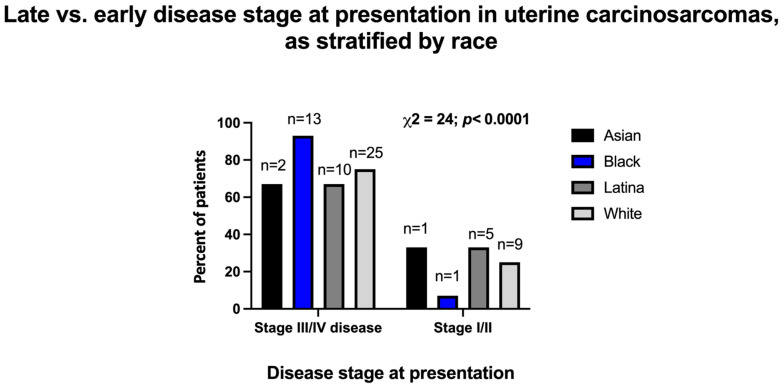
Early- vs. late-stage distribution upon presentation of patients with uterine carcinosarcoma, as stratified by the patients’ race. The bars represent percentages of Asian, Black, Latina, or White patients presenting at certain stages. Data were analyzed with chi-square test. Total number of uterine carcinosarcoma patients in this cohort is 66.

The majority of patients were diagnosed with advanced-stage disease at the time of presentation. At the time of their diagnosis, 50 patients with UCS (76%) and 14 patients with OCS (88%) had stage III or IV disease. The single case of CCS was stage II at diagnosis. When considering race/ethnicity, patients self-identifying as minorities (Black/African American, Hispanic/Latina, and Asian/Pacific Islander) with uterine carcinosarcoma were more likely to present with advanced-stage disease (stage III/IV) (*p* < 0.0001, df = 24). This difference was largely driven by the number of Black/African American patients presenting with advanced-stage disease: 93% of Black/African American patients, 74% of White patients (25/34), 67% of Hispanic/Latina patients, and 67% of Asian/Pacific Islander patients with UCS presented with advanced stage disease (Figure 1A). A total of 100% of Asian/Pacific Islander patients, 92% of White patients, and 67% of Hispanic/Latina patients with OCS presented with advanced-stage disease (Figure 1B). Figure 2 depicts the comparison of women with uterine carcinosarcoma presenting with late-stage (III/IV) versus early-stage (I/II) disease.

### 3.3. Survival

Survival data are presented in Table 4. White patients had a median PFS of 0 months and a median OS of 11.5 months. The median PFS and OS in Black/African American patients were 9 months and 13 months, respectively. In Hispanic/Latina patients, the median PFS was 7 months, and the median OS was 13 months. Asian/Pacific Islander patients had a median PFS of 5.5 months, and a median OS of 18 months.

The one-year survival in all patients was 55%, with 45% five-year survival in all patients. In White patients, one- and five-year survival was 54.4% and 45.7%, respectively. Of White patients, 10.9% remained alive at the time of data collection. Black/African American patients had a one-year survival of 64.3% and a five-year survival of 43%, with 14.3% of patients alive at the time of data collection. A total of 52.3% of Latina/Hispanic patients were alive one year after diagnosis, with 42% surviving at five years and 10.5% alive at the time of data collection. In Asian/Pacific Islander patients, both one- and five-year survival rates were 50%, with 25% of patients alive at the time of data collection. The median PFS overall was 7.5 months and only 6.5 months in patients presenting with stage IV disease.

### 3.4. Treatment Course

Among patients with UCS, 43 (65.2%) patients underwent primary extirpative surgery. Of the remaining patients, 14 (21.2%) initially underwent pelvic radiation therapy, and 2 (3%) received chemotherapy as the initial course of treatment. Seven patients with UCS (10.6%) elected for supportive care only. Nine (52.9%) patients with OCS initially underwent surgical staging or debulking, four (23.5%) initially underwent radiation therapy, and two (11.8%) initially received chemotherapy; one patient was lost to follow up after diagnosis and treatment details are not available. The patient with CCS underwent surgery prior to any further treatment.

A total of 57 UCS patients (86.4%) received chemotherapy at any point during their treatment for carcinosarcoma. All patients with UCS received carboplatin-paclitaxel as their first regimen of systemic chemotherapy. Eleven OCS patients (68.8%) were treated with chemotherapy at some point during their treatment course. Nine OCS patients were initially treated with carboplatin-paclitaxel, one was treated with Doxil-cisplatin-ifosfamide, and one patient received chemotherapy with an outside provider for which records are not available. Four patients (one with ovarian and three with uterine cancer) were treated with ifosfamide-paclitaxel as second-line chemotherapy after initially progressing on carboplatin-paclitaxel. Two additional patients received ifosfamide-based regimens, including the OCS patient initially treated with an ifosfamide-based regimen. No patients included in this cohort were treated with a trastuzumab-containing regimen at any point during their treatment course. 

### 3.5. Molecular Characterization

Beginning in 2016, immunohistochemistry (IHC) testing for mismatch repair (MMR) deficiency was performed on all uterine cancer diagnoses at our institution. This was extended to cases of ovarian carcinosarcoma as well. A total of 22 tumors were tested for MMR deficiency, 19 UCS and 3 OCS. Out of the 22 tested, 1 UCS case was noted to be deficient in mismatch repair (MLH1 and PMS2). No cases of OCS were noted to be MMR-deficient. This represents a total rate of MMR deficiency of 4.5% among all patients tested and a rate of 5.3% among UCS cases tested.

Of the patients studied here, only one, a patient with stage I ovarian carcinosarcoma at time of diagnosis, underwent tumor testing for HER2/neu expression. Her tumor demonstrated HER2/neu amplification. 

Ten patients underwent germline genetic testing to evaluate for hereditary cancer syndromes: three were patients with carcinosarcoma arising in the ovary (3/16 OCS patients, 18.8%) and seven with uterine carcinosarcoma (7/66 UCS patients, 10.6%). Of these, one OCS patient was found to have a pathogenic mutation in *BRCA1* gene, and one UCS patient was determined to carry a variant of unknown significance (VUS) in the *BRCA2* gene. The remaining eight patients tested for hereditary cancer syndromes were not found to carry any pathologic mutations.

## 4. Discussion

In the current investigation, the rate of UCS was 5.2% (66/1278 patients) in a cohort of patients treated for endometrial cancer at a single tertiary care institution in southern California. The patient population presented in this study represents a more diverse group of patients than has previously been reported, with 44.6% of patients included in the study self-identifying as a minority (Black/African American, Hispanic/Latina, and Asian/Pacific Islander). Across all cases of carcinosarcoma, minority patients were more likely to present with advanced-stage disease (stage III/IV) at the time of diagnosis than patients identifying as White. This difference was largely driven by the high percentage of Black/African American patients presenting to care with advanced-stage disease. The vast majority (93%) of patients identifying as Black/African American presented with advanced-stage disease. In contrast, we found that fewer Hispanic/Latina and Asian/Pacific Islander patients presented with advanced-stage disease compared with patients identifying as White. The majority of Hispanic/Latina and Asian/Pacific Islander patients also presented with advanced-stage disease. Factors contributing to gynecologic healthcare disparities in minority populations include less education, lower rates of healthcare literacy, higher unemployment rates, and lack of healthcare-provider-initiated referrals [16]. Previous studies in other gynecological cancer processes (i.e., addressing cervical cancer screening in minority women) have suggested that increased rates of poverty and lower rates of health insurance contribute to disparities in screening rates [16]. The same disparities may be applicable to the current population of our study.

In addition to the classic association between obesity- and estrogen-driven type 1 endometrial cancers, obesity has also been associated with poor prognosis in patients with carcinosarcoma [17]. In the United States, Black/African American and Latina/Hispanic women are more likely to be overweight or obese compared with White women [18]; while not statistically analyzed, due to statistical power restrictions, these factors likely contributed to the poor prognosis identified in this study.

The 5.2% rate of uterine carcinosarcoma in this study is slightly higher than the previously reported rate of <5% of all uterine malignancies. The rates of ovarian and cervical carcinosarcoma found here are consistent with those of prior reports. There was a noticeable drop in the rates of both uterine and ovarian carcinosarcoma in 2019 and 2020, and although the rate of UCS remained lower in 2021, in 2021, the rate of OCS was higher than in all the prior years except for 2013. The COVID-19 pandemic, beginning in 2020, was a possible contributing factor to this decrease, as there were redistributions and changes in the utilization pattern of health resources. Many patients had limited access to preventive healthcare services and were less likely to present for care other than that needed for COVID-19-related illnesses and complications. This may have led patients with gynecologic issues to delay seeking care, as demonstrated by the lower census of gynecologic cancer diagnoses overall during this period. We note that prior to 2019, the annual rates of UCS were higher than prior reports—it remains to be seen if we will see a return to similar rates in the years following the acute phase of the COVID-19 pandemic. We also note that the COVID-19 pandemic does not explain the lower rates of carcinosarcoma seen in 2019, which is likely secondary to the rarity of the disease.

Even in the era where carboplatin-paclitaxel is front-line chemotherapy, the median PFS of 7.5 months in all patients regardless of the disease site demonstrates the poor prognosis associated with the diagnosis. The aggressive nature of the disease is the likely reason for such short survival overall. It was previously thought that, like other gynecologic sarcomas, the sarcomatous component is responsible for the aggressive nature of carcinosarcomas. However, studies have demonstrated that lymphatic metastasis represents the dominant route of spread, similar to other variants of endometrial carcinoma [6]. In contrast, sarcomas are more likely to spread via hematogenous dissemination [6]. This association suggests that it is the epithelial component driving the aggressiveness of carcinosarcomas and is consistent with the current prevailing theory that uterine carcinosarcoma is a metaplastic carcinoma as opposed to a true sarcoma [6]. Despite this pathologic characterization, the prognosis for carcinosarcoma is significantly worse than for other aggressive endometrial histologies, including FIGO grade 3 endometrioid adenocarcinoma, uterine papillary serous carcinoma, and uterine clear cell carcinoma [19].

Molecular characterization of tumors has become the standard of care in gynecologic cancers in the past decade. The NCCN recommends testing all endometrial cancers for deficiencies in MMR genes [10]. Among all patients tested for MMR deficiency at our institution, only 4.5% were found to be deficient, and only 5.3% of patients with uterine carcinosarcoma were found to be MMR deficient. Although we do not have data available regarding the rates of MMR deficiency in all women with endometrial carcinoma at our institution, studies have found that 20–30% of patients with endometrial cancer are deficient in the mismatch repair pathway [20,21,22]. The rate of MMR deficiency demonstrated here is significantly lower than the previously published rates for endometrial cancer, and this deserves further study for uterine carcinosarcoma. 

The NCCN guidelines recommend genetic testing for all women diagnosed with ovarian cancer [11], as 20% of patients with ovarian cancer will be identified as carrying a pathogenic mutation [23]. Patients belonging to racial and ethnic minorities are less likely to complete this recommended genetic testing [24,25,26]. The rates of genetic testing in the population studied here were low, with only 10 patients undergoing testing (12.0% of patients). Among patients with ovarian carcinosarcoma, 18.8% of patients completed genetic testing. These low rates of testing may be explained in part by the large numbers of racial and ethnic minority patients in this patient population. Only two patients tested were found to have a mutation in a *BRCA* gene (20%), and one of these was a patient with uterine carcinosarcoma, who was found to have a variant of unknown significance in the *BRCA2* gene, for a final pathogenic mutation rate of 10%. The one patient with a pathogenic mutation in *BRCA1* had ovarian carcinosarcoma, a pathogenic mutation rate of 33% among ovarian carcinosarcoma patients tested. However, this is likely to be falsely elevated by the low rates of completion of genetic testing, as only three patients with ovarian carcinosarcoma completed genetic testing.

In recent years, improved molecular characterization has furthered the development and testing of targeted therapies [27,28]. Recent studies have demonstrated improved outcomes in patients with advanced and recurrent endometrial cancer treated with immunotherapy in combination with traditional cytotoxic chemotherapy [29,30]. Although the rate of mismatch repair (MMR) deficiency was low in this cohort, recent studies have demonstrated the benefit of immunotherapy even in patients with proficient mismatch repair. The recent RUBY trial included 49 patients with UCS; however, it did not include a subgroup analysis of patients with carcinosarcoma, although it demonstrated improvement in both PFS and OS in patients with histologies other than endometrioid that were treated with dostarlimab, carboplatin, and paclitaxel [29]. As we enter the era of frontline immunotherapy for endometrial cancer, we are hopeful this will lead to improved outcomes in patients with UCS.

Although inherent to the rarity of this diagnosis, we recognize that the small sample size is a limitation of this study. The strengths of this study include the centralized pathology review and a single comprehensive cancer center, the diversity of the patient population studied, and the ability to gather more comprehensive data due to the full availability of electronic medical records as compared with the population-based database studies commonly utilized for the study of this rare diagnosis.

## 5. Conclusions

We found that the rate of UCS was >5% of all endometrial malignancies in a 10-year longitudinal cohort. We found that women of racial and ethnic minorities are significantly more likely to present with advanced-stage disease at the time of diagnosis with carcinosarcoma compared with patients identifying as White. This discrepancy is likely multifactorial but warrants further study to examine possible areas that may be targeted to improve early diagnosis and outcomes. Carcinosarcoma is an aggressive cancer, and even in cases at an early stage, the average PFS is only 18 months. Gaps in our understanding of carcinosarcoma may be attributed to its low incidence and complex biphasic histology resulting in poor reproducibility of diagnosis among pathologists. As molecular testing and immunotherapies demonstrating improved outcomes in endometrial and cervical cancer become the standard of care, we expect these therapeutics to be studied and used in the treatment of carcinosarcoma as well, hopefully leading to improved outcomes in patients with carcinosarcoma.

## Figures and Tables

**Table 1:** Carcinosarcoma cases by type at a single institution from January 2012 to May 2021.

Cancer Type	Total Number of Diagnoses (N)	Carcinosarcoma Diagnoses N (Percentage of Total)
Uterine	1278	66 (5.2%)
Ovarian	476	17 (3.6%)
Cervical	362	1 (0.28%)
All Patients	2116	84 (4.0%)

**Table 2:** Carcinosarcoma incidence by year and type.

	2012	2013	2014	2015	2016	2017	2018	2019	2020	2021
Uterine	6.5%	7.5%	6.0%	5.0%	6.6%	6.0%	5.4%	1.4%	1.7%	4.1%
Ovarian	3.3%	9.4%	2.0%	1.6%	4.4%	2.0%	3.1%	2.7%	0%	5.0%
Cervical	-	-	-	-	-	-	-	2.5%	-	-

**Table 3:** Summary of patients diagnosed with gynecologic carcinosarcoma at a single institution from January 2012 to May 2021.

Demographic Characteristic	Uterine 66 (79.5%)	Ovarian 16 (19.3%)	Cervical 1 (1.2%)	Total 83
Median Age (years)	69	59	58	68
BMI				
Underweight (BMI < 20)	4 (6.1%)	1 (6.3%)	--	5 (6.0%)
Normal (BMI 20–<25)	15 (22.7%)	7 (43.8%)	--	22 (26.5%)
Overweight (BMI 25–<30)	18 (27.3%)	4 (25%)	--	22 (26.5%)
Obese (BMI 30–35)	19 (28.8%)	--	1 (100%)	20 (24.1%)
Severe Obesity (BMI > 35)	3 (4.5%)	4 (25%)	--	7 (8.4%)
Morbid Obesity (BMI > 40)	7 (10.6%)	--	--	7 (8.4%)
Race/Ethnicity				
White	34 (51.5%)	12 (75%)	--	46 (55.4%)
Black/African American	14 (21.2%)	--	--	14 (16.9%)
Hispanic/Latina	15 (22.7%)	3 (18.8%)	1 (100%)	19 (22.9%)
Asian/Pacific Islander	3 (4.5%)	1 (6.3%)	--	4 (4.8%)
Menopausal Status				
Premenopausal	4 (6.1%)	2 (12.5%)	--	6 (7.2%)
Postmenopausal	61 (92.4%)	13 (81.3%)	1 (100%)	75 (90.4%)
Stage at Diagnosis				
Stage I	16 (24.2%)	1 (6.3%)	--	17 (20.5%)
Stage II	--	1 (6.3%)	1 (100%)	2 (2.4%)
Stage IIIA	8 (12.1%)	1 (6.3%)	--	9 (10.8%)
Stage IIIB	3 (4.5%)	1 (6.3%)	--	4 (4.8%)
Stage IIIC	18 (27.3%)	11 (68.8%)	--	29 (34.9%)
Stage IV	16 (24.2%)	1 (6.3%)	--	17 (20.5%)
Unstaged/Staging Unavailable	5 (7.6%)	--	--	5 (6.0%)

**Table 4:** Survival data by race/ethnicity for all patients with carcinosarcoma.

Race/Ethnicity	Median Progression Free Survival (mos)	Median Overall Survival (mos)	One Year Survival (%)	Five Year Survival (%)	Overall Survival to Date (%)
White	0	11.5	55%	45%	10.9%
Black/African American	9	13	64.3%	43%	14.3%
Hispanic/Latina	7	13	52.3%	42%	10.5%
Asian/Pacific Islander	5.5	18	50%	50%	25%

## Data Availability

The data presented in this study are available on request from the corresponding author. The data are not publicly available due to privacy restrictions.
